# Exploring the Role of Anatomical Imaging Techniques in Preoperative Planning for Orthopaedic Surgeries

**DOI:** 10.7759/cureus.46622

**Published:** 2023-10-07

**Authors:** Kush D Patel, Dushyant D Desai, Jaymin K Bhatt, Dinesh R Patel, Vidya K Satapara

**Affiliations:** 1 Anesthesiology, Nootan Medical College and Research Center, Visnagar, IND; 2 Pathology, Nootan Medical College and Research Center, Visnagar, IND; 3 Pathology and Laboratory Medicine, Nootan Medical College and Research Center, Visnagar, IND; 4 Pathology, Nootan Medical College and Research Center, Nootan General Hospital, Visnagar, IND; 5 Anatomy, Gujarat Medical Education and Research Society (GMERS) Medical College, Gandhinagar, IND

**Keywords:** magnetic resonance imaging, computed tomography, magnetic imaging resonance, knee arthroplasty, anatomical imaging technique

## Abstract

Introduction: The incorporation of a three-dimensional (3D) framework enables the surgeon to strategically plan their surgical intervention through the utilisation of the printed model. This encompasses the process of ascertaining the surgical approach, choosing the suitable reduction technique, finding the required implant dimensions, defining the ideal placement and alignment of the implant, and conducting a simulated practise of the procedure using a 3D printed duplicate of the anatomical structures. Therefore, we designed this study to evaluate the role of two imaging modalities (computed tomography (CT) and magnetic resonance imaging (MRI)) for pre-surgical planning for orthopaedic surgeries.

Methodology: The present investigation entailed a prospective analysis of total knee arthroplasties (TKAs) that were performed using patient-specific instrumentation (PSI) from 2019 to 2022. After performing the bone resection operation utilising a customised cutting jig specific to each patient, the exact thickness of the resected bone was evaluated using a vernier calliper. In the MRI group, the researchers directly compared the cutting thickness during surgery with the consistency planned before the operation. In contrast, the CT group added the presumed cartilage thickness (2 mm) to the actual thickness of the bone that was removed from the lateral condyles.

Results: The planned incision thickness in the distal femoral was 8.5 ± 0.8 in the CT group and 8.9 ± 0.5 in the MRI group, while the actual incision thickness was reported as 9.8 ± 0.54 in CT and 8.3 ± 1.1; however, no significant mean difference was found between both groups. The planned incision thickness was 2.6 ± 1.1 in the CT group and 2.43 ± 1.66 in the MRI group, while the actual thickness was observed as 2.5 ± 0.6 and 2.88 ± 1.12 without significant difference (p = 0.86).

Conclusion: While magnetic resonance imaging (MRI) allows for the visualisation of cartilage, it has been observed that the MRI-based patient-specific instrumentation (PSI) system does not exhibit superior accuracy in projecting bone incision thickness compared to the computed tomography (CT)-based PSI system.

## Introduction

Pre-surgical planning has continually drawn much attention and interest in orthopaedic fracture surgery [1]. The significance of pre-surgical planning in orthopaedic fracture surgery should not be underestimated, given the variety of surgical techniques at our disposal and the crucial nature of attaining accurate rotation, axis alignment, and restoration of normal anatomical structures [2]. Recently, there have been noteworthy progressions in preoperative planning, particularly in templating technology [3]. The primary objective of preoperative planning is to enhance comprehension of the fracture, hence augmenting the likelihood of achieving a successful reduction and optimising osteosynthesis [3].

The discipline of orthopaedics has witnessed significant progress in the practical application of three-dimensional (3D) printing technology, which has undergone remarkable advancements [4]. There has been a considerable spike in the implementation of 3D printing technology within the field of orthopaedic surgery [4]. Nevertheless, the use of this technique within the field of orthopaedic surgery is still restricted, principally due to a deficient comprehension of its integration [5]. Currently, the global accessibility of 3D printing technology is constrained in numerous locations [3].

The three-dimensional printing technique facilitates the production of precise copies of anatomical structures obtained from computed tomography (CT) and magnetic resonance imaging (MRI) images [5,6]. The employment of these replicas offers significant benefits to surgeons since they facilitate preoperative planning and the practising of complicated orthopaedic surgery [7]. Incorporating 3D printing technology holds considerable significance in augmenting preoperative surgical planning within orthopaedics, specifically in instances that require intricate visualisation of three-dimensional anatomy and precise determination of implant dimensions for use during the surgical procedure [8]. The application of technology allows for the creation of preoperative 3D models that accurately replicate the anatomical and pathological features encountered by surgeons during intraoperative operations [9]. The incorporation of a three-dimensional (3D) framework enables the surgeon to strategically plan their surgical intervention through the utilisation of the printed model [10]. This technique encompasses ascertaining the surgical approach, choosing the suitable reduction technique, finding the required implant dimensions, defining the ideal placement and alignment of the implant, and conducting a simulated procedure practice using a 3D-printed duplicate of the anatomical structures [11]. The above characteristics empower the physician to make preoperative judgements on numerous surgical elements using 3D printing technology [10]. Utilising three-dimensional (3D) printing technology within the medical field offers many benefits. These include the potential to shorten surgical procedures, minimise the need for fluoroscopy, lower the risk of infection and implant misalignment, minimise the risk of blood loss during surgery, and alleviate various postoperative complications [3,7-11]. Hence, the incorporation of 3D printing technology improves the surgeon's capacity to precisely comprehend the three-dimensional alignment of anatomical components, a critical factor in guaranteeing the effective implementation of surgical interventions and enhancing the standard of patient care. Therefore, we designed this study to evaluate the role of two imaging modalities (CT and MRI) for pre-surgical planning for orthopaedic surgeries. The primary objective of this study was to determine whether the utilisation of MRI- or CT-based patient-specific instrumentation (PSI) could effectively replicate precise bone excision and yield improved postoperative results. Consequently, our study aimed to investigate whether the magnetic resonance imaging (MRI)-based patient-specific instrumentation (PSI), which accurately represents the cartilage layer, offers superior accuracy in total knee arthroplasty (TKA) compared to the computed tomography (CT)-based PSI. In this study, it was postulated that the MRI-based PSI would exhibit greater accuracy in determining the resection thickness of the lateral aspect of the proximal tibia, specifically in cases where the cartilage remains relatively intact in the varus knee.

## Materials and methods

Methodology

We conducted this study in the orthopaedic department of Nootan Medical College and Research Centre, Visnagar, Gujarat from 2019 to 2022. The present investigation entailed a prospective analysis of total knee arthroplasties (TKAs) that were performed using patient-specific instrumentation (PSI) under the guidance of a single surgeon (XYZ). 

Ethical approval

Ethical approval was granted by the institutional ethical committee of Nootan Medical College and Research Centre, Visnagar, Gujarat, for the study protocol (EC number: IEC/NMCRC/APPROVAL/06/2019).

Inclusion and exclusion criteria

The study included patients scheduled for total knee arthroplasty (TKA) to treat primary osteoarthritis with varus deformity. The study comprised just those individuals who had been awaiting total knee arthroplasty (TKA) for six weeks and had provided their informed consent to undergo the relatively innovative approach of TKA utilising a patient-specific instrumentation (PSI) system based on either magnetic resonance imaging (MRI) or computed tomography (CT). 

Sample size calculations and randomisation of the study

During the study duration, 94 patients were eligible for the study; however, only 71 participants provided their written consent. A four-block computer-based randomisation table was formed to randomly allocate the 75 participants of total knee arthroplasty to either the magnetic resonance imaging (MRI) group (N = 38) or the computed tomography (CT) group (N = 37). The subsequent evaluation was carried out two years post-surgery in all patients without any exceptions. The Signature™ Personalised Patient Care System, which was manufactured utilising Materialise® software (ISO environment with CE and FDA 510k) was employed in all cases involving patients.

Pre-surgical evaluation

The pre-surgical evaluation of group one was conducted through an MRI scanner (1.5 T MRI PHILIPS) provided by Nootan Medical College and Research Centre, Visnagar, Gujarat. High-resolution images of the knee (1 mm) and 5 mm spot images of the hip and ankle were obtained to measure the mechanical axis (MA) and correct any rotation in the lower extremities. The digital imaging and communications in medicine (DICOM)-formatted MRI scans were uploaded to the software platform, where three-dimensional (3D) models were generated. Following this, the cutting jigs were produced for each patient, utilising the predetermined default parameters given by the surgeon. 

On the other hand, before surgery, the CT scan (Siemens 128-slice CT scan) was done using the slices of the lower extremities, with a thickness of 1.25 mm, acquired to assess the mechanical axis (MA) and address any rotational misalignments in the lower extremity. The CT images, initially in DICOM format, were subjected to processing using the same software to produce three-dimensional models. The initial parameters for the MRI-based patient-specific instrumentation (PSI) in the CT group were set to replicate the approaches utilised for the medial bone cut. After the preoperative plan was authorised, the manufacturer of the patient-specific instrumentation (PSI) was instructed to create cutting jigs and bone models that accurately depict the anatomical structure of the patient's femur and tibia. The bone models and cutting jigs were made via fast prototyping techniques, with polyamide being the material for their construction.

Operation technique

The surgical procedures were conducted with the administration of general anaesthesia and the use of a tourniquet to regulate blood flow. These procedures were carried out by an experienced surgeon utilising a minimally invasive mid-vastus approach. The utilisation of patient-specific cutting jigs was employed for bone resection in all knee cases. Osteophyte removal was not conducted during this process. The cutting jigs were precisely positioned on both the femur and the tibia, guaranteeing a secure fit without any movement. The distal femur resection was carried out using a modified gap approach. Following this, the cutting of the proximal tibial bone and the resection of the femoral chamfer were conducted using PSI cutting jigs. The bone incisions were performed using conventional saw blades with a thickness of 1.27 mm. The posterior-stabilized cemented full knee system was administered to all participants. The employed approaches, including the insertion of a suction drain, utilisation of a pneumatic pump for deep vein thrombosis prevention, implementation of multimodal pain management strategies, and adherence to a postoperative rehabilitation protocol, were consistently administered to all patients.

Postoperative follow-up

After performing the bone resection operation utilising a customised cutting jig specific to each patient, the exact thickness of the resected bone was evaluated using a vernier calliper. The measurements were recorded at intervals of 0.1 mm. These measurements were then compared to the preoperative projected resection thickness determined by the PSI program. In the MRI group, the researchers directly compared the cutting thickness during surgery with the consistency planned before the operation. In contrast, the CT group added the presumed cartilage thickness (2 mm) to the actual thickness of the bone that was removed from the lateral condyles [12,13]. In all instances, the thickness of the saw blade (1.27 mm) was uniformly incorporated into the resected bone.

The clinical assessment was done before the surgery and at two years post-surgery in both groups. The knee scores of all the participants were evaluated using the guidelines described by Knee Society Scores [14]. A clinical examination was observed via a short survey containing 36 items developed by Ware and colleagues [15].

Data measurement and analysis

Data were gathered and analysed by using IBM Statistical Package for Social Sciences (SPSS) version 23.0 (IBM SPSS Inc., Armonk, NY). We compared categorical variables by using Fisher's exact test. Meanwhile, the skewness of the data was tested, and a comparison was drawn using the Mann-Whitney test. Furthermore, the normally distributed data was compared by the student's t-test. The interclass correlation coefficient was used to investigate the inter- and intra-observer probabilities. The International Classification Codes (ICCs) were categorised into three groups based on their values: poor, which encompassed values below 0.4; marginal, which included values ranging from 0.4 to 0.75; and good, which comprised values above 0.75.

## Results

In the computed tomography group, 31 females and seven males were enrolled with a mean age of 67.2 ± 4.8 years, while 34 females and four males with a mean age of 68.2 ± 6.9 years were recruited in the magnetic resonance group. Before surgery, the femorotibial angle was -3.7 ± 4.2°, while the mechanical axis was reported as -9.6 ± 4.5° in the CT group. Meanwhile, the MRI group had a mechanical axis of -10.6 ± 4.4 (Table 1). The planned incision thickness in distal femoral was 8.5 ± 0.8 in the CT group and 8.9 ± 0.5 in the MRI group, while the actual incision thickness was reported as 9.8 ± 0.54 in CT and 8.3 ± 1.1 in MRI; however, no significant mean difference was found between both groups. The planned incision thickness in tibial medial side was 2.6 ± 1.1 in the CT group and 2.43 ± 1.66 in the MRI group, while the actual thickness was observed as 2.5 ± 0.6 and 2.88 ± 1.12 without significant difference (p = 0.86). A detailed analysis was shown in Table 2. Table 3 depicts the mean difference in actual and planned incision thickness at distal femoral medial side, tibial medial side, and distal femoral lateral side. The intraobserver intraclass correlation coefficient (ICC) ranged from 0.883 to 0.945 for each variable, while the interobserver ICC ranged from 0.817 to 0.8263

**Table 1:** Demographic characteristics and clinical outcomes before surgery. Mechanical axis (°), femorotibial angle (°).

Parameters	Computed tomography (N = 37)	Magnetic resonance imaging (N = 38)	p-value
Age (mean ± SD)	67.2 ± 4.8	68.2 ± 6.9	0.59
Sex			0.28
Male	7 (18.9%)	4 (10.5%)	
Female	31 (83.7%	34 (89.4%)	
BMI (kg/m^2^) (mean ± SD)	26.9 ± 3.9	28.6 ± 3.1	0.89
Femorotibial angle before surgery (mean ± SD)	–3.7 ± 4.2	–3.9 ± 4.8	0.43
Mechanical axis before surgery (mean ± SD)	–9.6 ± 4.5	–10.6 ± 4.4	0.32

**Table 2:** Comparison of actual and planned thickness.

Parameters	Computed tomography (N = 37)	Magnetic resonance imaging (N = 38)	p-value
Distal femoral medial side (mean ± SD)			0.16
Planned	8.5 ± 0.8	8.9 ± 0.5	
Actual	9.8 ± 0.54	8.3 ± 1.1	
Tibial medial side (mean ± SD)			0.86
Planned	2.6 ± 1.1	2.43 ± 1.66	
Actual	2.5 ± 0.6	2.88 ± 1.12	
Distal femoral lateral side (mean ± SD)			0.19
Planned	9.1 ± 1.4	8.8 ± 0.99	
Actual	8.1 ± 1.6	7.99 ± 1.2	
Posterior femoral medial side (mean ± SD)			0.09
Planned	8.5 ± 0.3	9.0 ± 0.3	
Actual	8.7 ± 1.5	9.6 ± 1.1	
Posterior femoral lateral side (mean ± SD)			0.06
Planned	8.4 ± 0.99	7.8 ± 1.3	
Actual	6.9 ± 1.23	7.2 ± 1.1	
Tibial lateral side (mean ± SD)			0.69
Planned	9.9 ± 0.3	9.9 ± 0.2	
Actual	9.2 ± 1.2	9.2 ± 0.99	

**Table 3:** Mean difference in actual and planned thickness in both groups.

Parameters	Magnetic resonance imaging (mean ± SD)	Computed tomography (mean ± SD)
Distal femoral medial side	0.8 ± 0.6	1.1 ± 0.6
Tibial medial side	0.98 ± 0.67	1.1 ± 0.7
Distal femoral lateral side	1.3 ± 0.7	1.5 ± 1.0
Posterior femoral medial side	0.9 ± 0.7	1.3 ± 0.8
Posterior femoral lateral side	1.2 ± 0.78	1.6 ± 0.7
Tibial lateral side	1.1 ± 0.6	1.2 ± 0.7

There was no statistically significant disparity observed in the average preoperative and postoperative Knee Society knee and functional scores, as well as the 36-item Short Form Survey, between the two groups at the two-year mark following the surgical procedure. No reoccurrence of the incident was observed in the two-year follow-up, and not even a single patient required re-operation. Moreover, there were no perioperative problems noted in the study, such as deep or superficial infections, symptomatic deep vein thrombosis or pulmonary embolisms, and periprosthetic fractures, until the latest follow-up assessment (Table 4).

**Table 4:** Clinical outcomes before and after surgery.

Parameters	Computed tomography (N = 37)	Magnetic resonance imaging (N = 38)	p-value
Physical function score (SF-36)			
Before surgery	39.8 ± 7.9	29.7 ± 9.6	0.68
At two-year follow-up	47.3 ± 7.9	46.9 ± 7.9	0.97
Knee Society function score			
Before surgery	45.6 ± 9.6	46.5 ± 9.5	0.15
At two-year follow-up	88.4 ± 11.9	90.4 ± 14.3	0.46
Knee Society knee score			
Before surgery	41.5 ± 15.5	43.8 ± 17.5	0.39
At two-year follow-up	92.1 ± 10.8	91.7 ± 9.8	0.56

## Discussion

The emergence of the 3D printing technique in the orthopaedic field has shown promise in several surgical operations related to the knee. This technology has provided phenomenal results, such as total knee arthroplasty (TKA) [4]. Traditionally, knee arthroplasty was conducted using plain radiography (X-rays) to determine the appropriate component size and predict the postoperative alignment of the limb [16]. However, introducing a 3D printing approach for total knee arthroplasty combined with the patient-specific cutting guide provided better accuracy rates for bone alignment. The current study investigated the role of 3D technology (MRI and CT) in preoperative planning for total knee arthroplasty. For this purpose, the two groups underwent MRI and CT examinations separately to assess the outcomes. Overall, similar mean values of clinical outcomes were observed in both imaging modalities (CT and MRI), along with similar rates of bone alignment after surgery. Traditionally, surgeons used fixed criteria of valgus femur angle for proximal TKA despite the variations in angles of individuals [16]. However, patient-specific three-dimensional technology helps surgeons suggest the bone-cutting technique during surgery. Nowadays, these techniques vary from patient to patient for both femoral and tibial knee replacements [17]. The study's inclusion criteria expressly mandated the existence of a primary osteoarthritic knee with varus deformity as the primary disease. Therefore, it was hypothesised that there was a significant level of degeneration in the medial femoral cartilage, although the thickness of the cartilage in the lateral tibial condyle remained relatively preserved in all participants included in the research. Within the framework of the CT group, an extra 2 mm of estimated cartilage thickness was integrated into the lateral tibial resection bone thickness. The aforementioned modification was implemented in order to accommodate the existence of the cartilage layer around the subchondral bone, which was not discernible by CT imaging. No modifications were deemed essential in the MRI group, as the MRI technique demonstrated a high level of accuracy in depicting the cartilage layer. The findings of the study indicated that there was no statistically significant difference between the two groups in terms of the amount of bone removal on the outer side of the thigh bone.

In the current study, the intended and actual incision thickness for the distal femur was similar in both groups. Similar results were found for the proximal tibial; however, images obtained from the CT group failed to visualise the cartilage layers on the subchondral bone; therefore, we added a 2 mm estimated osseous thickness during the incision made on the lateral tibia. Meanwhile, no changes were necessary for the MRI group as MRI could accurately represent the cartilage layer. According to our findings, an insignificant difference was found for the area of bone resection on the lateral femur surface in both study groups. Hence, it is advisable to augment the cartilage thickness value by roughly 2 mm, as suggested by the CT-based PSI system, when evaluating the resection thickness of the lateral sides of the tibia in patients exhibiting varus deformity [12,13].

A wide range of studies have been conducted to compare the effectiveness of MRI and CT for measuring the distal femur and tibial incision thickness in total knee arthroplasty [18-20]. The majority of the studies used the PSI system to validate their results [18-20]. Recently, a clinical trial found that MRI-based PSI provides more accuracy for the coronal mechanical axis of the limb than CT-based PSI [18]. However, it is essential to note that the observed changes were relatively slight, and their clinical significance remains uncertain [18].

Both MRI and CT imaging techniques have distinct advantages and disadvantages when employed in the development of a patient-specific implant (PSI) system [19]. However, a disparity will inevitably arise between the bone model and the dimensions of the patient's femur and tibia due to variations in measuring and manufacturing procedures [21]. In knee arthroplasty, magnetic resonance imaging (MRI)-based patient-specific instrumentation (PSI) offers the benefit of providing a comprehensive view of the cartilage layer; however, CT imaging demonstrates superior accuracy when determining the planned incision thickness [21,22]. In the present investigation, an insignificant difference was observed among both study groups when comparing the average coronal and sagittal alignments of the femoral and tibial components. Nevertheless, our analysis found no distinction between the MRI and CT groups across all three planes. 

Study limitations

The present study also exhibited several shortcomings. First, this study does not measure the cost of the PSI system. Secondly, it is worth noting that the sample size of subjects in the study was quite limited. The present study was designed with a prospective approach; nevertheless, the determination of the sample size was not based on a thorough investigation or calculation. Lastly, an assessment of the PSI compared to traditional instruments in total knee arthroplasty (TKA) was not conducted. Furthermore, radiation exposure and cost-effectiveness were not observed. Therefore, we recommend conducting a comparative study between the MR/CT-based 3D model group and a group where 3D models weren't used at all to prove the superiority of this exercise in clinical scenarios.

## Conclusions

While magnetic resonance imaging (MRI) allows for the visualisation of cartilage, it has been observed that the MRI-based patient-specific instrumentation (PSI) system does not exhibit superior accuracy in projecting bone incision thickness compared to the computed tomography (CT)-based PSI system. Furthermore, no significant disparities were observed regarding radiographic results and clinical outcomes across both groups. There is a need for further studies to evaluate the cost and resource requirements for both approaches; therefore, we recommended more research.

## References

[1] Wade RH, Kevu J, Doyle J (1998). Pre-operative planning in orthopaedics: a study of surgeons' opinions. Injury.

[2] Liu B, Luo X, Huang R, Wan C, Zhang B, Hu W, Yue Z (2014). Virtual plate pre-bending for the long bone fracture based on axis pre-alignment. Comput Med Imaging Graph.

[3] Krakowski P, Karpiński R, Maciejewski M (2018). Applications of modern imaging technology in orthopaedic trauma surgery. Appl Comput Sci.

[4] Duan X, Wang B, Yang L, Kadakia AR (2021). Applications of 3D printing technology in orthopedic treatment. Biomed Res Int.

[5] Haleem A, Javaid M (2018). Role of CT and MRI in the design and development of orthopaedic model using additive manufacturing. J Clin Orthop Trauma.

[6] Duncan JM, Nahas S, Akhtar K, Daurka J (2015). The use of a 3D printer in pre-operative planning for a patient requiring acetabular reconstructive surgery. J Orthop Case Rep.

[7] Hung CC, Li YT, Chou YC, Chen JE, Wu CC, Shen HC, Yeh TT (2019). Conventional plate fixation method versus pre-operative virtual simulation and three-dimensional printing-assisted contoured plate fixation method in the treatment of anterior pelvic ring fracture. Int Orthop.

[8] Xiao JR, Huang WD, Yang XH (2016). En bloc resection of primary malignant bone tumor in the cervical spine based on 3-dimensional printing technology. Orthop Surg.

[9] Zhang YD, Wu RY, Xie DD, Zhang L, He Y, Zhang H (2018). Effect of 3D printing technology on pelvic fractures: a meta-analysis. Zhongguo Gu Shang.

[10] Wu ZX, Huang LY, Sang HX, Ma ZS, Wan SY, Cui G, Lei W (2011). Accuracy and safety assessment of pedicle screw placement using the rapid prototyping technique in severe congenital scoliosis. J Spinal Disord Tech.

[11] Van Genechten W, Van Tilborg W, Van den Bempt M, Van Haver A, Verdonk P (2021). Feasibility and 3D planning of a novel patient-specific instrumentation technique in medial opening-wedge high tibial osteotomy. J Knee Surg.

[12] Bardakos N, Cil A, Thompson B, Stocks G (2007). Mechanical axis cannot be restored in total knee arthroplasty with a fixed valgus resection angle: a radiographic study. J Arthroplasty.

[13] Deakin AH, Basanagoudar PL, Nunag P, Johnston AT, Sarungi M (2012). Natural distribution of the femoral mechanical-anatomical angle in an osteoarthritic population and its relevance to total knee arthroplasty. Knee.

[14] Martimbianco AL, Calabrese FR, Iha LA, Petrilli M, Lira Neto O, Carneiro Filho M (2012). Reliability of the "American Knee Society Score" (AKSS). Acta Orthop Bras.

[15] Ware JE Jr, Sherbourne CD (1992). The MOS 36-item short-form health survey (SF-36). I. Conceptual framework and item selection. Med Care.

[16] Kladny B, Bail H, Swoboda B, Schiwy-Bochat H, Beyer WF, Weseloh G (1996). Cartilage thickness measurement in magnetic resonance imaging. Osteoarthritis Cartilage.

[17] Shepherd DE, Seedhom BB (1999). Thickness of human articular cartilage in joints of the lower limb. Ann Rheum Dis.

[18] Pfitzner T, Abdel MP, von Roth P, Perka C, Hommel H (2014). Small improvements in mechanical axis alignment achieved with MRI versus CT-based patient-specific instruments in TKA: a randomized clinical trial. Clin Orthop Relat Res.

[19] Silva A, Pinto E, Sampaio R (2016). Rotational alignment in patient-specific instrumentation in TKA: MRI or CT?. Knee Surg, Sports Traumatol, Arthrosc.

[20] Asada S, Mori S, Matsushita T, Nakagawa K, Tsukamoto I, Akagi M (2014). Comparison of MRI- and CT-based patient-specific guides for total knee arthroplasty. Knee.

[21] White D, Chelule KL, Seedhom BB (2008). Accuracy of MRI vs CT imaging with particular reference to patient specific templates for total knee replacement surgery. Int J Med Robot Comput Assist Surg.

[22] Tibesku CO, Innocenti B, Wong P, Salehi A, Labey L (2012). Can CT-based patient-matched instrumentation achieve consistent rotational alignment in knee arthroplasty?. Arch Orthop Trauma Surg.

